# Homeostatic Correction During Severe Physiologic Stress: A Phase-Specific Model for GC–GRα-Coordinated Organism-Wide Adaptation

**DOI:** 10.3390/medsci14060638

**Published:** 2026-10-04

**Authors:** Gianfranco Umberto Meduri, Antoni Torres

**Affiliations:** 1 Department of Medicine and Pharmaceutical Sciences, University of Tennessee Health Science Center, Memphis, TN 38163, USA; 2 Institut d’Investigacions Biomédiques August Pi i Sunyer (IDIBAPS), University of Barcelona, CIBERES, 08036 Barcelona, Spain; atorres@recerca.clinic.cat; 3 Centro de Investigación Biomédica en Red de Enfermedades Respiratorias (CIBERES), 28029 Madrid, Spain

**Keywords:** homeostatic correction, adaptive capacity, glucocorticoid receptor alpha (GRα), glucocorticoid signaling, critical illness, systems biology, physiologic resilience, inter-organ communication, precision medicine, homeostasis

## Abstract

Critical illness is increasingly recognized as a disorder of disrupted systemic homeostasis rather than simply a consequence of maladaptive systemic inflammation or isolated organ failure. Although researchers have extensively investigated adaptive biologic responses to severe physiologic stress, far less attention has been paid to the integrative mechanisms that coordinate these responses across multiple organ systems throughout the course of illness. This review synthesizes current evidence from systems biology, immunology, endocrinology, network physiology, and critical care to present a systems-level conceptual model of homeostatic correction that explains how coordinated adaptive biologic programs restore organismal homeostasis after severe physiologic stress. The model organizes these responses into three dynamic, partially overlapping phases: Priming, which establishes adaptive readiness and immediate survival responses; Modulatory, which limits excessive activation while preserving essential host-defense and physiologic functions; and Restorative, which promotes resolution, tissue repair, regenerative recovery, and the restoration of integrated organ-system function. In this model, glucocorticoid–glucocorticoid receptor alpha (GC–GRα) signaling serves as a central integrative regulator coordinating immune, neuroendocrine, metabolic, mitochondrial, vascular, epithelial, lymphatic, microbiome, and regenerative adaptive biologic programs through context-, tissue-, and phase-specific mechanisms. Rather than acting solely as an anti-inflammatory pathway, GC–GRα signaling functions as a dynamic, systems-level regulator coordinating adaptive capacity, inter-organ communication, disease tolerance, tissue protection, and recovery while preserving essential host-defense functions. The proposed model shifts the focus from isolated pathophysiologic abnormalities to understanding critical illness as a disorder of disrupted organism-wide adaptive coordination. It provides a biologically organized foundation for investigating adaptive capacity, identifying phase-specific biomarkers, and developing precision therapeutic strategies to restore homeostatic resilience across acute and chronic disease states.

## 1. Introduction

Life depends on the ability to continuously maintain internal stability while adapting to an ever-changing environment. Throughout evolution, multicellular organisms have developed highly integrated surveillance, communication, and response systems that continuously detect external and internal threats and coordinate adaptive biologic responses that preserve survival, tissue integrity, and organismal function [1,2,3]. Whether the initiating stimulus is infection, trauma, ischemia, toxic exposure, metabolic disturbance, or psychological stress, successful adaptation depends not only on the activation of individual defense mechanisms but also on the coordinated regulation of multiple interconnected biologic systems operating throughout the entire organism [4,5,6].

Understanding how multicellular organisms maintain homeostasis during severe physiologic stress requires a conceptual model that integrates diverse adaptive biologic systems into a coordinated, organism-wide response. Current understanding of severe physiologic stress has advanced substantially through studies of individual components of the host response, including innate and adaptive immunity, inflammation, neuroendocrine regulation, metabolism, mitochondrial bioenergetics, endothelial and epithelial barrier function, lymphatic biology, the microbiome, tissue repair, and regenerative mechanisms [7,8,9,10]. These investigations have greatly expanded our understanding of the molecular and cellular pathways involved in adaptation to injury and disease. Nevertheless, these biologic systems are generally studied as largely independent processes, despite extensive evidence that they function as highly interconnected adaptive networks whose activities must be continuously coordinated across space and time [11,12,13].

An effective adaptive response requires considerably more than recognizing danger or activating inflammation. It requires the rapid mobilization of appropriate defense mechanisms, continuous recalibration of those responses as physiologic conditions evolve, and efficient termination of adaptive programs once homeostasis has been restored [4,7,14]. Failure of any component of this integrated adaptive process—including inadequate activation, excessive amplification, impaired coordination, delayed resolution, or incomplete restoration—may contribute to persistent inflammation, immune dysfunction, metabolic instability, organ failure, chronic critical illness, inflammaging, progressive loss of adaptive resilience, and the development or progression of chronic disease [15,16,17].

This concept may be particularly relevant to early recognition of sepsis. Emerging longitudinal evidence indicates that biologic changes may precede overt organ dysfunction, with unfavorable trajectories characterized by concurrent innate immune activation and adaptive immune suppression, metabolic and mitochondrial alterations, and endothelial injury [18,19,20,21]. These findings suggest that progression toward sepsis may be recognized not by a single biomarker or isolated abnormality, but by serial detection of an adverse trajectory across multiple interconnected adaptive systems [22]. Although routinely available clinical and laboratory measurements may provide early evidence of such progression, more specific immune, endothelial, metabolic, mitochondrial, and transcriptomic markers remain investigational. Prospective studies that integrate these measurements in the same patients are needed to determine whether emerging failure of homeostatic correction can be identified early enough to permit intervention before clinically apparent organ dysfunction develops.

These observations suggest that a broader conceptual model is needed to explain how the diverse adaptive biologic systems responsible for host defense and tissue protection are integrated into a coordinated organism-wide response. Rather than functioning as isolated pathways, immune, neuroendocrine, metabolic, vascular, mitochondrial, lymphatic, microbiome, and regenerative systems communicate continuously through complex molecular, cellular, endocrine, neural, and metabolic signaling networks that collectively preserve physiologic homeostasis and adaptive resilience.

This article presents homeostatic correction as an integrative conceptual model for understanding organism-wide adaptation during severe physiologic stress. Within this model, adaptation is a dynamic process that progresses through three partially overlapping phases—the Priming Phase, the Modulatory Phase, and the Restorative Phase—each characterized by distinct yet highly coordinated biologic objectives [23]. Across these phases, glucocorticoid (GC)–glucocorticoid receptor alpha (GRα) signaling—the integrated sequence of glucocorticoid production, receptor activation, intracellular signal transduction, and context-dependent regulation of gene expression and cellular function—serves as a central integrative regulator that helps coordinate the magnitude, timing, tissue specificity, and duration of adaptive responses across multiple organ systems, thereby promoting successful restoration of homeostasis and physiologic resilience.

Because the adaptive response encompasses numerous interacting biologic systems, the present manuscript focuses on establishing the conceptual model rather than providing a comprehensive review of individual adaptive pathways. The companion manuscripts examine these systems in greater detail. The first Companion Manuscript [24] examines the foundational immune, metabolic, mitochondrial, and vascular adaptive programs that establish the earliest organism-wide responses to severe physiologic stress. The second Companion Manuscript [25] examines the complementary neuroendocrine, adaptive immune, lymphatic, barrier–microbiome, and regenerative systems that coordinate organism-wide integration, recovery, and restoration across the three phases of homeostatic correction.

Together, these three manuscripts present an integrated systems-level model that explains how coordinated adaptive biologic programs preserve survival, restore homeostasis, and promote long-term physiologic resilience. Across these manuscripts, the model describes the coordinated interactions among multiple biologic systems that communicate through interconnected signaling networks, generating integrated organism-wide adaptive responses that cannot be fully understood by examining individual organs or signaling pathways in isolation.

## 2. Literature Search and Evidence Selection

This narrative review was informed by structured, hypothesis-driven literature searches conducted from July through September 2026. Consensus served as the primary artificial intelligence-assisted platform for structured literature discovery and evidence retrieval. Searches were supplemented by PubMed/MEDLINE and Google Scholar, reference-list review, and targeted retrieval of relevant primary and review articles. Search concepts included glucocorticoids, glucocorticoid receptor alpha (GRα), hypothalamic–pituitary–adrenal axis, critical illness, severe physiologic stress, inflammation, innate and adaptive immunity, immunometabolism, mitochondrial function, endothelial and vascular homeostasis, inter-organ communication, lymphatic function, microbiome–gut interactions, epithelial-barrier integrity, tissue repair, regeneration, disease tolerance, resolution biology, and physiologic resilience.

We selected the literature based on relevance to the proposed framework, mechanistic and clinical importance, and its contribution to defining phase-specific or integrative adaptive responses. Priority was given to primary experimental, translational, and clinical studies, with authoritative reviews used where appropriate to provide broader mechanistic or conceptual context. Because this article aimed to develop and evaluate a systems-level conceptual framework rather than conduct a systematic review or meta-analysis, we did not undertake formal systematic-review procedures, quantitative evidence grading, or exhaustive study inclusion. The authors critically evaluated all of the retrieved literature and independently developed the conceptual framework, interpretation, synthesis, tables, figures, and conclusions.

## 3. Homeostatic Correction: An Integrative Model for Adaptive Capacity

### 3.1. Biological Basis of Adaptive Capacity

Adaptive capacity is the ability of an organism to detect physiologic disturbances, mobilize appropriate adaptive responses, maintain essential biologic functions during stress, and restore homeostasis after the insult resolves. This capacity depends not only on the integrity of individual biologic systems but also on their ability to communicate, coordinate, and continuously recalibrate their activities in response to changing physiologic conditions [26,27,28,29,30,31].

Across these disciplines, adaptive capacity depends on physiologic reserve, biologic plasticity—the capacity of cells, tissues, and organ systems to dynamically modify their structure, function, and interactions in response to changing physiologic demands—dynamic regulation, and coordinated communication among multiple biologic systems. Together, these properties enable the organism to continuously detect, respond, adapt, and recover as physiologic conditions evolve. Rather than reflecting the function of individual organs or isolated signaling pathways, adaptive capacity emerges from the integrated, dynamic coordination of interconnected immune, neuroendocrine, metabolic, vascular, mitochondrial, lymphatic, microbiome, and regenerative systems, whose collective activity enables effective organism-wide adaptation and the preservation of homeostasis [11,12,13,32,33,34].

Recent advances in systems biology and network physiology indicate that adaptive capacity depends on coordinated interactions among multiple biologic systems rather than on the function of individual organs or signaling pathways alone. These systems continuously communicate and adjust their activity as physiologic conditions change, enabling the organism to respond to stress while preserving overall function [11,12].

Collectively, these observations suggest that adaptive capacity is an emergent, organism-wide property arising from the coordinated function of multiple interacting biologic systems. However, existing concepts do not fully explain how these adaptive systems are dynamically integrated, coordinated, and recalibrated as physiologic demands shift during severe physiologic stress. The homeostatic correction model was developed to address this conceptual gap by providing a phase-specific framework for organism-wide adaptation, recovery, and the restoration of systemic homeostasis.

### 3.2. Homeostatic Correction

Homeostatic correction is the dynamic, organism-wide process by which the body adapts to severe physiologic stress and works to restore homeostasis. It involves detecting physiologic disturbances, integrating signals across organ systems, activating appropriate responses, continuously recalibrating as conditions evolve, and timely resolution of these responses as homeostasis is restored. Rather than a single signaling pathway or physiologic mechanism, homeostatic correction provides a functional framework for understanding how multiple adaptive biologic systems are coordinated over time to preserve survival, restore tissue integrity, re-establish systemic homeostasis, and support long-term physiologic resilience.

This concept extends beyond traditional models of homeostasis, which primarily emphasize maintaining relatively stable internal conditions, and complements allostasis, which describes achieving stability through adaptive physiological change. The effectiveness of homeostatic correction depends not only on activating individual adaptive responses but also on their temporal coordination, proportionality to the magnitude of the threat, and timely termination once the initiating disturbance has resolved. Failure of homeostatic correction may occur at multiple levels, resulting from inadequate activation of protective responses, impaired communication among adaptive systems, loss of proportional regulation, delayed restoration of physiologic function, or persistent activation even after the original danger has resolved. Such dysregulation may contribute to maladaptive systemic inflammation, immune dysfunction, metabolic derangements, endothelial injury, mitochondrial dysfunction, chronic critical illness, progressive loss of adaptive resilience, inflammaging, and the development or progression of chronic disease [8,9,17,35]. Accordingly, clinical outcomes may depend not only on the magnitude of the initial adaptive response but also on the organism’s capacity to recalibrate, redirect, and transition among adaptive programs as physiologic requirements evolve. Persistent failure to make these transitions is associated with chronic critical illness, impaired functional recovery, and mortality [36,37,38,39,40].

Homeostatic correction incorporates but is conceptually distinct from several established frameworks of physiologic adaptation. Homeostasis describes maintaining internal stability, whereas allostasis emphasizes achieving stability through adaptive physiologic change. Stress-adaptation models describe how biologic priorities change during sustained stress [41]; disease tolerance refers to mechanisms that limit tissue injury and preserve host fitness without necessarily reducing pathogen burden; resolution biology defines active programs that terminate inflammation and promote tissue repair; and physiologic resilience describes the capacity to withstand perturbation and recover function [42,43,44]. Homeostatic correction integrates these complementary processes within a broader temporal and systems-level framework that emphasizes how multiple adaptive biologic programs are coordinated and recalibrated across the partially overlapping Priming, Modulatory, and Restorative phases. Its distinctive contribution is therefore not to propose new individual biologic mechanisms but to organize them into a testable model of organism-wide adaptive coordination during severe physiologic stress.

The model predicts that successful recovery will be associated with coordinated longitudinal transitions across multiple biologic domains, whereas persistent, asynchronous, or discordant trajectories will characterize impaired homeostatic correction and adverse outcomes. These predictions can be tested through serial multidomain molecular, physiologic, and clinical measurements. The framework further predicts that therapeutic responsiveness may depend on the patient’s evolving biologic state and the phase-specific functional requirements of homeostatic correction, providing a rationale for investigating biomarker-informed, phase-appropriate therapeutic strategies rather than applying interventions uniformly across heterogeneous biologic states.

**Figure 1:**
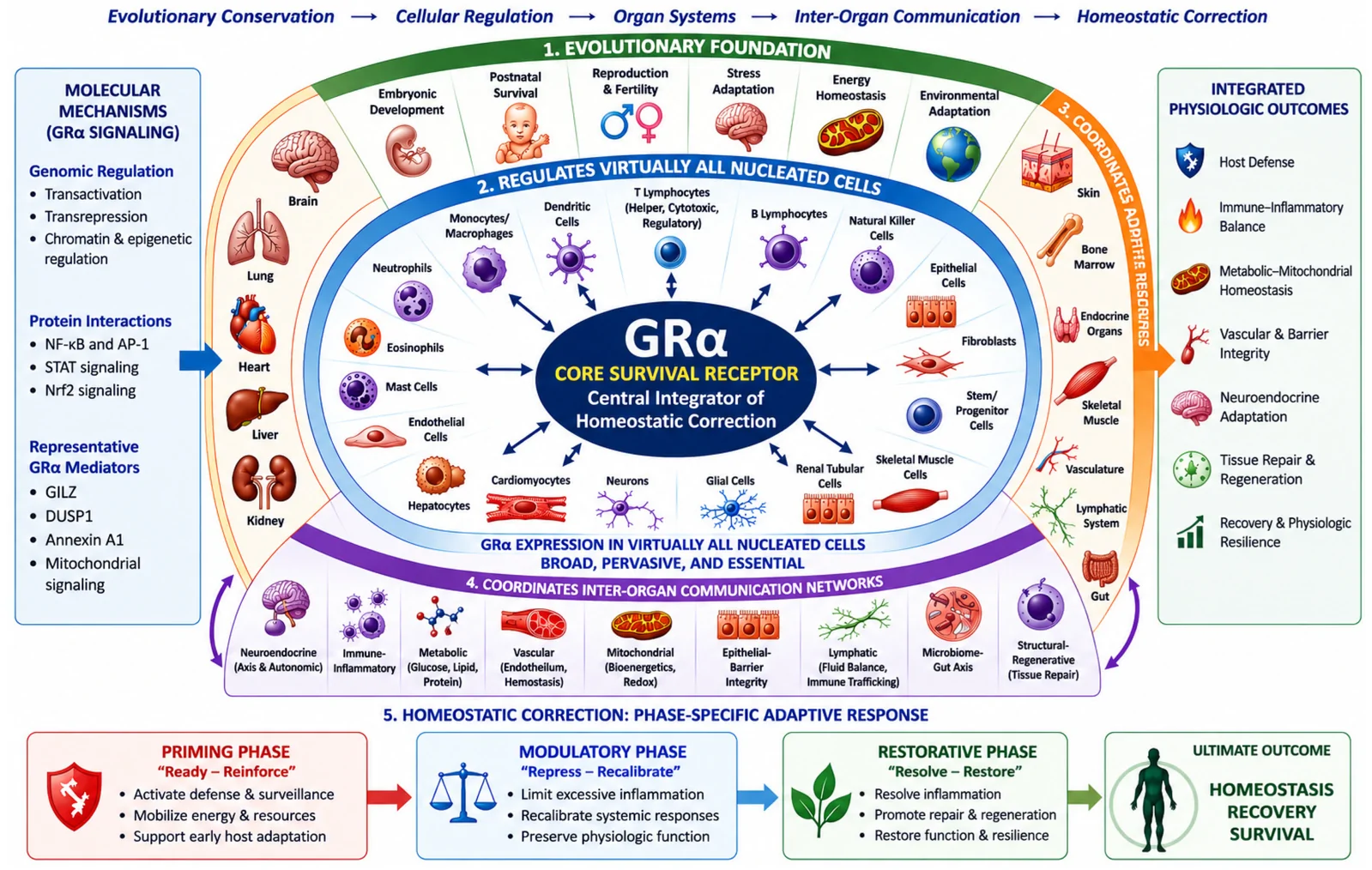
Glucocorticoid Receptor Alpha (GRα): A Core Survival Receptor and Central Integrative Regulator of Homeostatic Correction. The conceptual model illustrates glucocorticoid receptor alpha (GRα) as a core survival receptor and central integrator of homeostatic correction. GRα is broadly expressed across nucleated cells and regulates adaptive responses across organ systems through interconnected neuroendocrine, immune-inflammatory, metabolic, vascular, mitochondrial, epithelial-barrier, lymphatic, microbiome, and structural-regenerative networks. Bidirectional interactions emphasize continuous integration between GC–GRα signaling and these adaptive biologic systems. The lower portion depicts the three dynamic, partially overlapping phases of homeostatic correction—Priming (“Ready–Reinforce”), Modulatory (“Repress–Recalibrate”), and Restorative (“Resolve–Restore”)—through which evolving adaptive responses support restoration of homeostasis, recovery, and survival. Abbreviations: AP-1, activator protein-1; GC, glucocorticoid; GILZ, glucocorticoid-induced leucine zipper; GRα, glucocorticoid receptor alpha; NF-κB, nuclear factor kappa B; Nrf2, nuclear factor erythroid 2-related factor 2; STAT, signal transducer and activator of transcription. AI Acknowledgment: This figure was conceptually designed by the author. Artificial intelligence-assisted tools were used exclusively for graphical organization, visual refinement, layout optimization, color harmonization, and figure formatting. The authors developed, reviewed, and approved all scientific concepts, biologic interpretations, mechanistic models, and figure content.

Successful homeostatic correction requires coordinated regulation of adaptive responses across virtually every organ system, continuously adjusting their magnitude, timing, tissue specificity, and duration as physiologic conditions change. GC–GRα signaling fulfills this role by serving as a core survival receptor and a central integrator of organism-wide adaptation. Figure 1 summarizes the biologic properties that uniquely position GC–GRα signaling as a core survival receptor coordinating homeostatic correction. These properties include evolutionary conservation, broad expression across virtually all nucleated cells, organism-wide regulation of adaptive biologic programs, and integration with inter-organ communication networks. Together, these characteristics position GC–GRα signaling as a central integrator of coordinated adaptive responses that underlie homeostatic correction [3,23,33,45].

Because the organism’s adaptive requirements evolve continuously during severe physiologic stress, homeostatic correction is best understood as an adaptive process organized into three partially overlapping functional phases. Together, these phases provide a conceptual model for how integrated adaptive biologic programs are sequentially activated, coordinated, recalibrated, and ultimately resolved to restore organism-wide homeostasis. The following sections introduce these phases and describe how the principal biologic systems are coordinated throughout each phase to achieve effective homeostatic correction.

### 3.3. The Three Dynamic Phases of Homeostatic Correction

Current understanding holds that severe physiologic stress elicits an integrated, organism-wide host-response program rather than activating isolated inflammatory, endocrine, or metabolic pathways. Contemporary evidence shows that adaptive responses arise from coordinated interactions among immune, neuroendocrine, metabolic, mitochondrial, vascular, epithelial, microbiome, and tissue-repair systems, whose functional priorities shift continuously as physiologic conditions evolve [46,47,48,49,50,51,52,53].

This systems-level perspective provides the biologic rationale for organizing homeostatic correction into three dynamic, partially overlapping phases. As physiologic stress evolves, coordinated transitions are required from adaptive readiness to physiologic recalibration and ultimately to recovery and restoration [48,49,51,54].

The concept that the biological response to severe stress unfolds in temporally organized phases has deep experimental roots. Beginning with Selye’s experimental studies of the general adaptation syndrome (GAS) in the 1930s, stress adaptation was conceptualized as a dynamic progression through distinct stages [41,55]. Although the mechanisms and non-specificity proposed in the original model have since been substantially revised, the broader concept that adaptive priorities shift over the course of a sustained stress response has endured. The novelty of the present framework lies not in proposing temporally organized phases, but in redefining these responses as overlapping Priming, Modulatory, and Restorative adaptive programs and in integrating their coordinated operation across multiple biological systems during severe physiologic stress.

**Figure 2:**
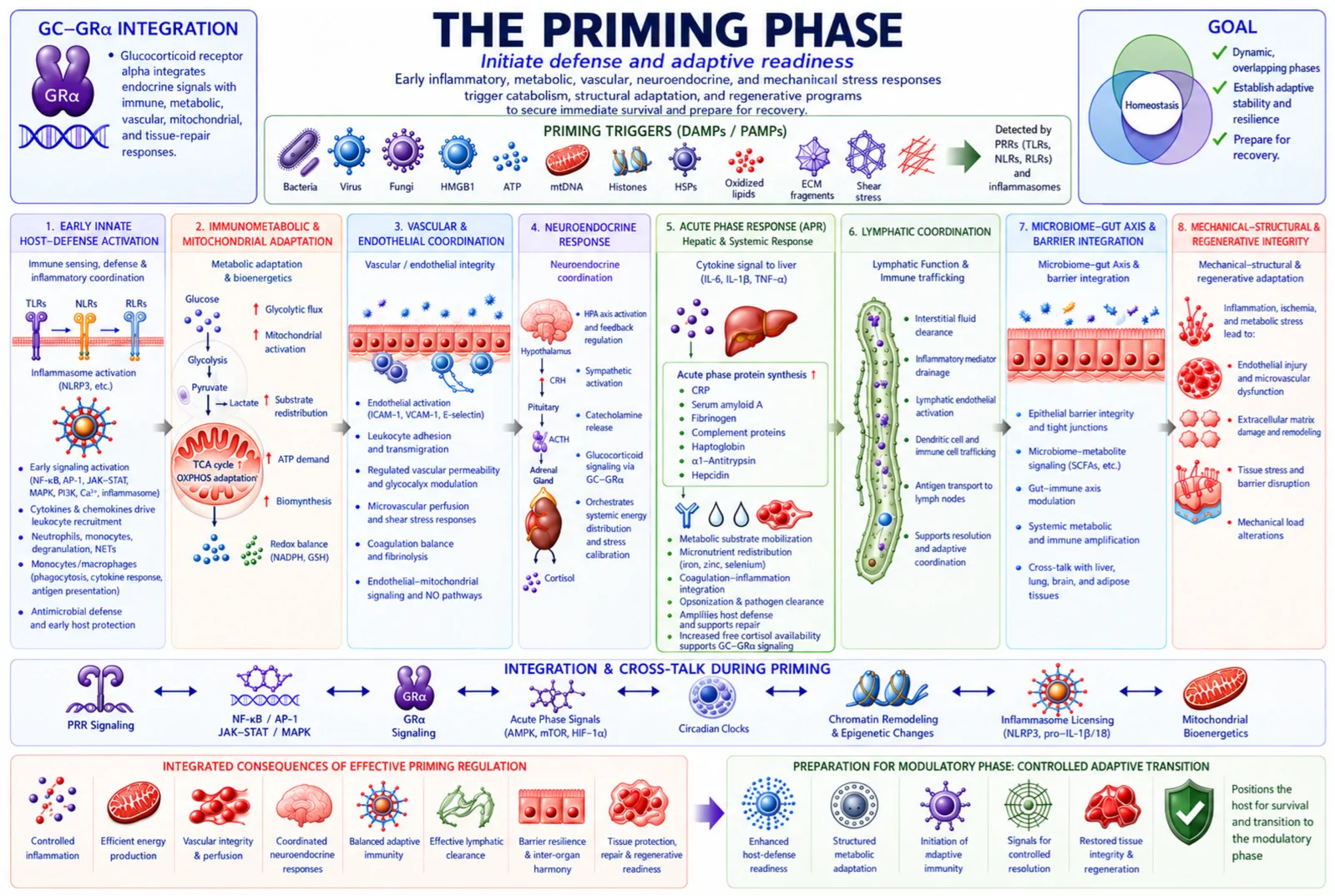
The Priming Phase: Initiation of Defense and Adaptive Readiness. This figure illustrates the Priming Phase of homeostatic correction during severe physiologic stress and critical illness. Infectious and noninfectious danger signals activate integrated inflammatory-defense, metabolic, vascular, neuroendocrine, hepatic acute-phase, mitochondrial, lymphatic, epithelial, and structural adaptive programs that support immediate survival and prepare the host for transition toward later modulatory and restorative phases. The figure is organized sequentially to reflect the biologic progression of the Priming Phase, including (1) innate immune sensing and inflammatory coordination, (2) immunometabolic and mitochondrial adaptation, (3) vascular and endothelial coordination, (4) neuroendocrine responses, (5) hepatic acute-phase and systemic metabolic responses, (6) lymphatic function and immune trafficking, (7) microbiome–gut and barrier–system interactions, and (8) mechanical–structural and regenerative adaptation. The newly integrated Acute Phase Response (APR) panel highlights the liver–immune–metabolic axis as an adaptive component of early host adaptation. Pro-inflammatory cytokines including IL-6, IL-1β, and TNF-α stimulate hepatic acute-phase protein synthesis, metabolic substrate mobilization, coagulation–inflammation integration, micronutrient redistribution, and amplification of early host-defense responses during severe physiologic stress. The lower integration panel highlights dynamic cross-talk among inflammatory signaling pathways, GC–GRα signaling, metabolic pathways, circadian regulation, chromatin remodeling, inflammasome activity, acute-phase responses, and mitochondrial bioenergetics. The lower outcome panels summarize the consequences of effective versus maladaptive priming responses and their influence on subsequent homeostatic correction, recovery, or progression to organ dysfunction. Abbreviations: ACTH, adrenocorticotropic hormone; AP-1, activator protein-1; APR, acute phase response; ATP, adenosine triphosphate; CRH, corticotropin-releasing hormone; DAMPs, damage-associated molecular patterns; ECM, extracellular matrix; GSH, glutathione; GRα, glucocorticoid receptor alpha; HMGB1, high-mobility group box 1; HPA axis, hypothalamic–pituitary–adrenal axis; HSPs, heat-shock proteins; ICAM-1, intercellular adhesion molecule-1; IFNs, interferons; IL, interleukin; JAK-STAT, Janus kinase-signal transducer and activator of transcription; MAPK, mitogen-activated protein kinase; mtDNA, mitochondrial DNA; mTOR, mechanistic target of rapamycin; NADPH, nicotinamide adenine dinucleotide phosphate; NETs, neutrophil extracellular traps; NF-κB, nuclear factor-kappa B; NLRs, NOD-like receptors; OXPHOS, oxidative phosphorylation; PAMPs, pathogen-associated molecular patterns; PI3K, phosphoinositide 3-kinase; PPP, pentose phosphate pathway; PRRs, pattern-recognition receptors; RLRs, RIG-I-like receptors; ROS, reactive oxygen species; SAA, serum amyloid A; TCA cycle, tricarboxylic acid cycle; TGF-β, transforming growth factor beta; TLRs, Toll-like receptors; TNF, tumor necrosis factor; VCAM-1, vascular cell adhesion molecule-1. AI Acknowledgment: This figure was conceptually designed by the author. Artificial intelligence-assisted tools were used exclusively for graphical organization, visual refinement, layout optimization, color harmonization, and figure formatting. The authors developed, reviewed, and approved all scientific concepts, biologic interpretations, mechanistic models, and figure content.

**Figure 3:**
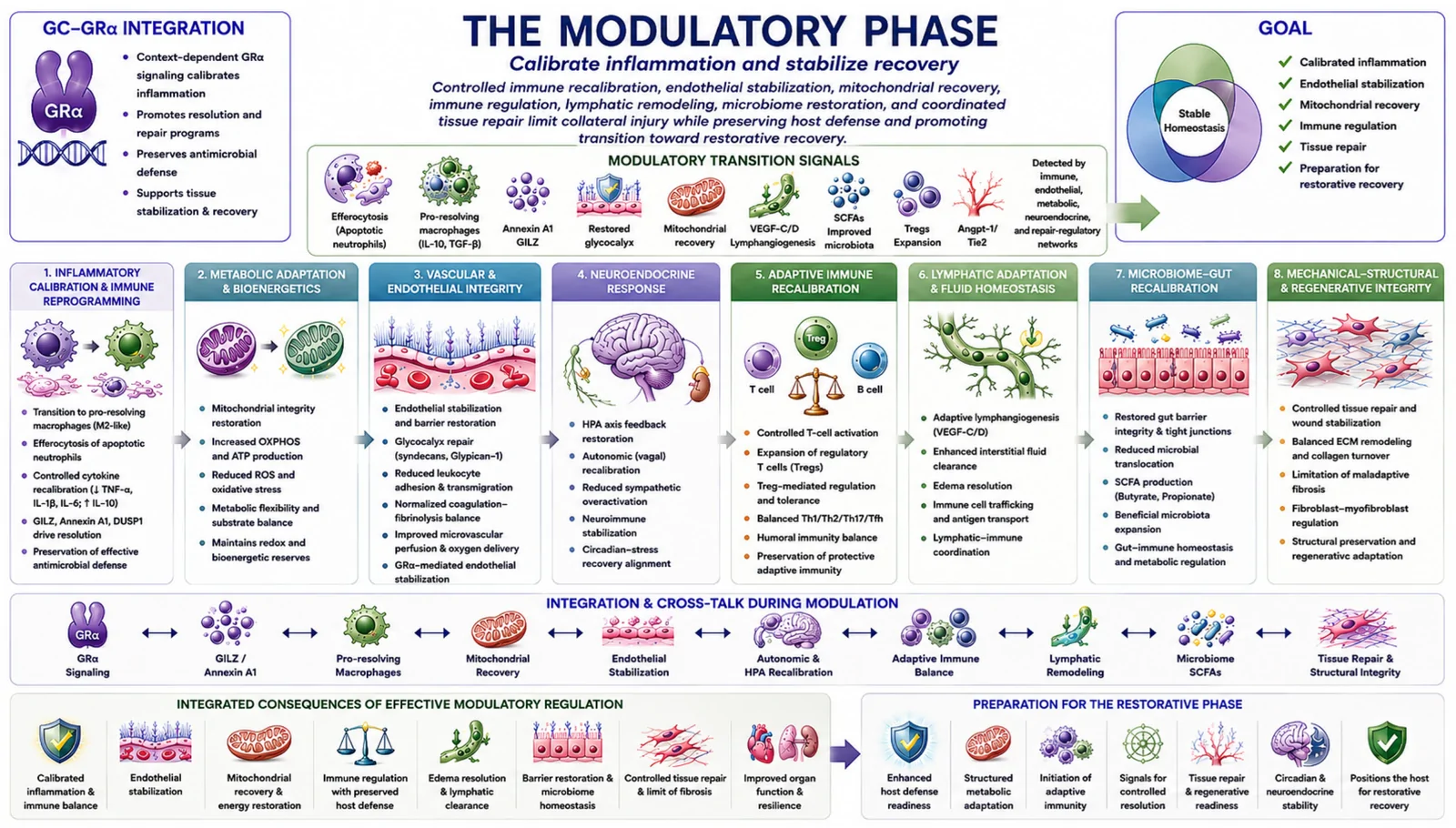
The Modulatory Phase: Physiologic Recalibration and Adaptive Stabilization. This figure illustrates the Modulatory Phase of homeostatic correction during severe physiological stress and critical illness. During this phase, early inflammatory-defense responses transition toward controlled immune recalibration, endothelial stabilization, mitochondrial recovery, lymphatic remodeling, microbiome restoration, and coordinated tissue-protective adaptation. Importantly, inflammatory activation and immune suppression are not mutually exclusive during critical illness; they may coexist, with persistent inflammatory signaling alongside impaired antigen presentation, lymphocyte dysfunction, regulatory-cell expansion, and immune exhaustion. Integrated interactions among GC–GRα signaling, immunometabolic regulation, vascular repair programs, neuroendocrine recalibration, adaptive immune coordination, lymphatic function, microbiome–gut interactions, and structural repair mechanisms limit collateral tissue injury, preserve antimicrobial competence, and promote progression toward restorative recovery. The figure is organized into interconnected biological domains that collectively coordinate adaptive stabilization during the Modulatory Phase, including (1) inflammatory calibration and immune reprogramming, (2) metabolic adaptation and mitochondrial recovery, (3) vascular and endothelial stabilization, (4) neuroendocrine recalibration, (5) adaptive immune regulation, (6) lymphatic remodeling and immune trafficking, (7) microbiome–gut barrier restoration, and (8) mechanical–structural and regenerative integrity. Bidirectional arrows indicate reciprocal cross-talk and functional interdependence among these biological domains, whereas directional arrows indicate progression of adaptive stabilization toward the Restorative Phase. Integrated signaling pathways involving GC–GRα, pro-resolving mediators, mitochondrial adaptation, endothelial repair programs, anti-inflammatory feedback regulation, and tissue-repair mechanisms collectively support restoration of systemic stability and preparation for transition toward the Restorative Phase. The lower integrated panels summarize the systems-level consequences of effective modulatory regulation, including calibrated inflammation, mitochondrial recovery, vascular stabilization, immune coordination, edema resolution, microbiome restoration, tissue repair, and enhanced organ resilience. The final transition panel illustrates how successful modulation restores systemic stability and prepares the host for the Restorative Phase, during which coordinated resolution, tissue repair, regeneration, and long-term recovery become the dominant biologic priorities. Abbreviations: ACTH, adrenocorticotropic hormone; Angpt-1, angiopoietin-1; ATP, adenosine triphosphate; DUSP1, dual-specificity phosphatase 1; ECM, extracellular matrix; GC, glucocorticoid; GRα, glucocorticoid receptor alpha; HPA, hypothalamic–pituitary–adrenal; IL, interleukin; OXPHOS, oxidative phosphorylation; SCFAs, short-chain fatty acids; TGF-β, transforming growth factor beta; Th, T helper cell; Treg, regulatory T cell; VEGF-C/D, vascular endothelial growth factor C/D. AI Acknowledgment: This figure was conceptually designed by the author. Artificial intelligence-assisted tools were used exclusively for graphical organization, visual refinement, layout optimization, color harmonization, and figure formatting. The authors developed, reviewed, and approved all scientific concepts, biologic interpretations, mechanistic models, and figure content.

**Figure 4:**
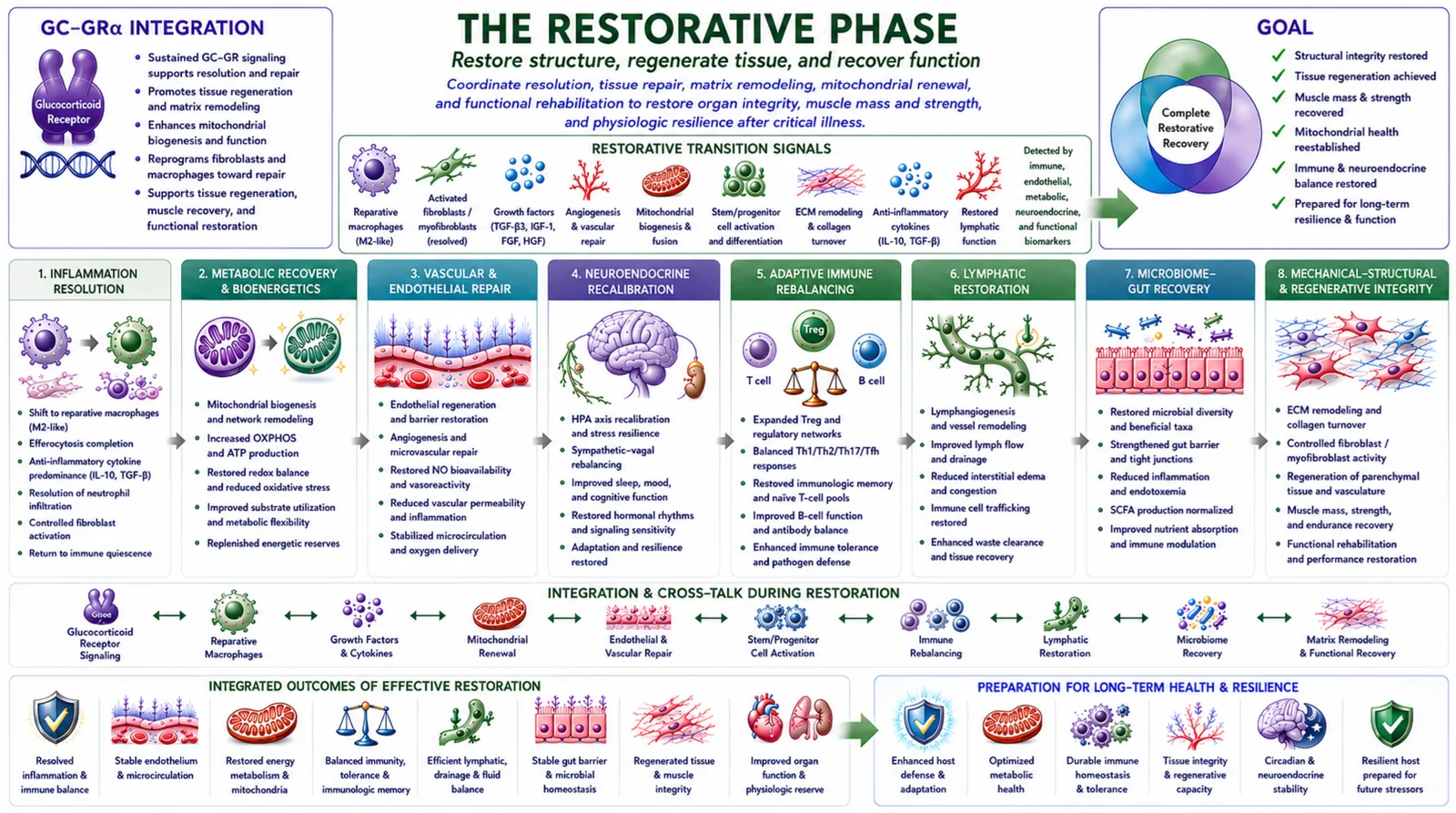
The Restorative Phase—Restore Homeostasis and Functional Recovery. This figure illustrates the Restorative Phase of homeostatic correction during severe physiologic stress and critical illness. Following the Priming and Modulatory Phases, integrated inflammatory-resolution, immune-regulatory, metabolic, mitochondrial, vascular, lymphatic, neuroendocrine, microbiome, and regenerative-repair programs cooperate to restore systemic homeostasis, tissue integrity, and long-term physiologic function. The figure is organized sequentially to reflect the biologic progression of restorative recovery, including (1) inflammatory resolution and reparative immune reprogramming, (2) mitochondrial recovery and metabolic restoration, (3) endothelial repair and microvascular stabilization, (4) neuroendocrine recalibration, (5) adaptive immune recovery and immune tolerance, (6) restoration of lymphatic architecture and immune trafficking, (7) microbiome–gut recovery and barrier restoration, and (8) mechanical–structural repair and regenerative remodeling. Bidirectional arrows indicate reciprocal cross-talk and functional interdependence among restorative processes, whereas directional arrows indicate progression toward integrated recovery, restored physiologic function, and long-term resilience. GC–GRα signaling is depicted as a central integrative regulator coordinating inflammatory resolution, mitochondrial adaptation, endothelial stabilization, immune recalibration, microbiome recovery, tissue repair, and regenerative homeostasis across organ systems. The integrated consequences of effective restorative regulation include recovery of tissue structure and function, restoration of immune and metabolic homeostasis, recovery of integrated organ-system function, and establishment of long-term physiologic resilience following severe physiologic stress. Abbreviations: ACTH, adrenocorticotropic hormone; AMPK, AMP-activated protein kinase; AP-1, activator protein-1; ATP, adenosine triphosphate; DAMPs, damage-associated molecular patterns; ECM, extracellular matrix; GC, glucocorticoid; GILZ, glucocorticoid-induced leucine zipper; GRα, glucocorticoid receptor alpha; HPA, hypothalamic–pituitary–adrenal; IL, interleukin; JAK–STAT, Janus kinase–signal transducer and activator of transcription; MAPK, mitogen-activated protein kinase; mtDNA, mitochondrial DNA; NETs, neutrophil extracellular traps; NF-κB, nuclear factor kappa-light-chain-enhancer of activated B cells; NLRs, nucleotide-binding oligomerization domain-like receptors; NO, nitric oxide; NLRP3, nucleotide-binding oligomerization domain leucine-rich repeat and pyrin domain-containing protein 3; OXPHOS, oxidative phosphorylation; PAMPs, pathogen-associated molecular patterns; PRRs, pattern-recognition receptors; RLRs, retinoic acid-inducible gene-I-like receptors; ROS, reactive oxygen species; SCFAs, short-chain fatty acids; TGF-β, transforming growth factor beta; TLRs, toll-like receptors; VEGF-C/D, vascular endothelial growth factor C/D; ZO-1, zonula occludens-1. AI Acknowledgment: This figure was conceptually designed by the author. Artificial intelligence-assisted tools were used exclusively for graphical organization, visual refinement, layout optimization, color harmonization, and figure formatting. The authors developed, reviewed, and approved all scientific concepts, biologic interpretations, mechanistic models, and figure content.

These programs are not rigid chronological compartments or synchronized organism-wide states. Their relative expression may vary across tissues, cellular populations, and biological domains and may proceed asynchronously within the same patient. Successful homeostatic correction therefore reflects coordinated progression across these adaptive programs, whereas incomplete, persistent, or discordant responses may lead to divergent trajectories of prolonged organ dysfunction, chronic critical illness, or death. Accordingly, the Priming, Modulatory, and Restorative Phases are characterized by distinct yet coordinated adaptive objectives and should be viewed as components of a single integrated adaptive process rather than as independent events. Figure 2, Figure 3 and Figure 4 illustrate, respectively, the principal biologic objectives, coordinated adaptive programs, and integrated outcomes of the Priming, Modulatory, and Restorative Phases [10,23,41,46,47].

Longitudinal studies of critically ill patients provide clinical support for this dynamic organization. Serial molecular and biomarker profiling shows that immune-inflammatory, immunoregulatory, metabolic, mitochondrial, and vascular/endothelial responses evolve concurrently rather than as isolated or strictly sequential processes. In ARDS, longitudinal transcriptomic profiling shows the time-dependent evolution of innate immune, metabolic-bioenergetic, vascular, and tissue-remodeling programs, with divergent molecular trajectories associated with survival [56]. Complementing these molecular observations, longitudinal inflammatory phenotyping demonstrates substantial within-patient transitions over the course of ARDS; nearly half of patients initially classified as hyperinflammatory transitioned to a hypoinflammatory phenotype within 30 days, and these transitions were strongly associated with clinical outcome. Large-scale proteomic profiling further identifies distinct multidomain biological states characterized by different combinations of innate immune activation, metabolic reprogramming, immune regulation, and tissue repair/remodeling, with inflammatory and reparative programs already concurrently activated early in ARDS [57]. Patients who recover rapidly show progressive recalibration across multiple biologic domains, whereas persistent multidomain dysregulation is linked to chronic critical illness, sustained organ dysfunction, hospital readmission, and mortality. Together, these observations support conceptualizing homeostatic correction as a dynamic process involving overlapping adaptive programs and biologic transitions rather than fixed phenotypes or strictly chronological intervals [36,37,39,58,59].

The homeostatic correction framework is intended to describe a common organizational architecture of the adaptive response rather than a uniform biologic program across all forms of severe physiologic stress. The proposed universal components include threat recognition and adaptive mobilization, subsequent recalibration of potentially injurious responses while preserving essential host functions, and resolution, repair, and restoration of physiologic homeostasis. In contrast, the magnitude, timing, duration, cellular composition, molecular pathways, and relative dominance of these adaptive programs are expected to vary substantially according to the initiating insult. Infectious challenges additionally require pathogen recognition, antimicrobial defense, and pathogen clearance, whereas sterile inflammatory, traumatic, ischemic, or metabolic insults engage distinct combinations of damage sensing, metabolic adaptation, tissue protection, and repair. Host characteristics further modify these responses, including age, genetic and epigenetic background, comorbidities, pre-existing immune and endocrine status, medications, nutritional and metabolic state, and prior environmental exposures. Thus, the Priming, Modulatory, and Restorative phases represent a shared organizational framework whose specific biologic expression is context-dependent rather than a universal sequence of identical molecular or cellular events.

#### 3.3.1. Priming Phase

The Priming Phase is the earliest stage of homeostatic correction and establishes adaptive readiness after severe physiologic stress. During this phase, coordinated activation of immune, neuroendocrine, metabolic, mitochondrial, vascular, epithelial, lymphatic, microbiome, and structural adaptive programs promote immediate survival while preparing the organism for subsequent physiologic recalibration. Figure 2 summarizes the primary biologic objectives, coordinated adaptive programs, and integrated outcomes that define the Priming Phase [23,60,61].

#### 3.3.2. Modulatory Phase

As the initiating threat is progressively controlled, the organism’s adaptive priorities shift toward physiologic recalibration and stabilization. During the Modulatory Phase, coordinated regulation of immune, metabolic, vascular, neuroendocrine, and reparative programs limits excessive tissue injury, preserves essential host-defense functions, and promotes disease tolerance. Figure 3 summarizes the principal biologic objectives, coordinated adaptive programs, and integrated outcomes that characterize the Modulatory Phase [10,54,62,63].

#### 3.3.3. Restorative Phase

The Restorative Phase is the final stage of homeostatic correction, marked by active resolution, tissue repair, regenerative recovery, and the restoration of integrated organ-system function. Rather than passive recovery, this phase coordinates immune, metabolic, vascular, mitochondrial, epithelial, lymphatic, microbiome, and regenerative adaptive programs to re-establish systemic homeostasis and long-term physiologic resilience. Figure 4 summarizes the principal biologic objectives, coordinated adaptive programs, and integrated outcomes that define the Restorative Phase [64,65,66,67,68]. Together, these three phases provide the organizational model for understanding how adaptive biologic programs are coordinated over time under severe physiologic stress [10,23].

The following sections examine the biologic principles underlying this model, while the companion manuscripts [24,25] describe, in greater detail, the phase-specific mechanisms by which GC–GRα signaling coordinates adaptive responses within individual biologic systems.

## 4. Biological Properties That Uniquely Position GC–GRα as a Central Integrative Regulator of Homeostatic Correction

The preceding sections demonstrate that effective homeostatic correction requires coordinated transitions among multiple biologic systems as adaptive priorities evolve during severe physiologic stress. This raises a fundamental biologic question: How are these complex, phase-specific adaptive responses coordinated across diverse tissues while remaining proportional to the nature, magnitude, and duration of the physiologic threat? Glucocorticoid (GC)–glucocorticoid receptor alpha (GRα) signaling—the integrated neuroendocrine-to-cellular communication system through which glucocorticoids activate GRα to regulate context-dependent transcriptional, metabolic, mitochondrial, vascular, immune, and reparative responses—serves as a central integrative regulator that helps coordinate the magnitude, timing, tissue specificity, and duration of adaptive responses across multiple organ systems, thereby promoting successful restoration of homeostasis and physiologic resilience.

This concept is supported by a series of complementary investigations demonstrating GC–GRα function as a core survival receptor, its coordination of phase-specific homeostatic correction during critical illness, its regulation of mitochondrial, endothelial, and intestinal homeostasis, and its integration of inter-organ communication networks [23,33,45,69].

Collectively, these investigations demonstrate that GRα is expressed in virtually all nucleated cells, regulates adaptive functions across every major organ system and circulating immune-cell population, and participates in extensive inter-organ communication networks that contribute to the maintenance and restoration of systemic homeostasis [23,33,45,69].

Rather than reviewing these mechanisms in detail, the present review builds upon this scientific foundation to examine how these unique biologic properties enable GC–GRα signaling to coordinate the phase-specific adaptive programs of homeostatic correction.

### Biological Basis for GC–GRα Coordination of Homeostatic Correction

Unlike most signaling systems that primarily regulate individual biological pathways or specific tissues, GC–GRα signaling exhibits a distinctive combination of biologic properties that collectively enable the coordinated regulation of organism-wide adaptive responses during severe physiological stress. These properties include ubiquitous cellular expression, endocrine accessibility to virtually all organs, context-dependent regulation of cell-specific transcriptional programs, integration of genomic and non-genomic signaling, coordination of nuclear and mitochondrial function, and the capacity to synchronize adaptive responses across multiple interacting organ systems while preserving tissue-specific functional specialization [23,33,45,69,70,71,72,73]. The biological and mechanistic properties that collectively support the proposed role of GC–GRα as a central integrative regulator of organism-wide homeostatic correction are summarized in Table 1 [3,33,66,69,74,75,76,77,78,79]. Importantly, organism-wide integration does not imply uniform GRα activity across tissues or an exclusive or hierarchical regulatory role. Rather, systemic glucocorticoid signaling generates context-dependent cellular responses shaped by tissue-specific receptor state, chromatin accessibility, co-regulators, local signaling networks, and metabolic conditions. GC–GRα is therefore positioned within a distributed network of interacting adaptive systems.

Independent experimental and translational evidence further supports this integrative role. In macrophages, GC–GR signaling directly couples inflammatory regulation to cellular metabolism and mitochondrial function, with glucocorticoid-induced transcriptional responses dependent in part on mitochondrial bioenergetic activity [80]. In human critical illness and complementary experimental models, GRα activity is dynamically and differentially regulated across immune cells and peripheral tissues, indicating that systemic glucocorticoid signaling does not elicit uniform cellular responses but instead generates tissue-specific adaptive programs in response to local physiologic requirements [66,81]. Together, these observations support a model in which organism-wide GC–GRα integration emerges through coordinated, context-dependent regulation across biologic systems rather than through uniform receptor activity across tissues.

The capacity of GC–GRα signaling to coordinate adaptive responses during critical illness also depends on receptor availability and functional sensitivity, which may vary across cell types and over time. Alterations in GR expression, isoform balance, post-translational modification, nuclear translocation, co-regulator availability, and interactions with inflammatory signaling pathways may produce tissue- or cell-specific glucocorticoid resistance or altered responsiveness, including within immune-cell populations. Such changes could modify the magnitude and timing of GR-mediated responses across the Priming, Modulatory, and Restorative phases and contribute to asynchronous or impaired homeostatic correction. In parallel, critical illness may disrupt normal HPA-axis regulation, including altered ACTH–cortisol relationships, changes in cortisol metabolism and availability, and impaired feedback regulation, further modifying endogenous GC–GR signaling. Importantly, the physiologic actions of endogenous GC–GRα signaling should be distinguished from the pharmacologic effects of exogenous corticosteroid therapy. Although both engage GR-dependent pathways, pharmacologic corticosteroids differ in dose, timing, receptor exposure, tissue distribution, and pharmacokinetics and therefore should not be considered biologically equivalent to endogenous stress-responsive GC–GRα signaling.

The biologic significance of this organization extends beyond broad tissue distribution. Because endogenous glucocorticoids circulate systemically, activation of the hypothalamic–pituitary–adrenal axis allows a single neuroendocrine stress signal to rapidly reach virtually every nucleated cell in the body. However, the resulting biologic response is not uniform. Instead, GRα generates highly context-dependent, cell-specific transcriptional programs determined by chromatin accessibility, transcription-factor availability, transcriptional co-regulators, epigenetic modifications, local signaling networks, metabolic state, and the temporal phase of the adaptive response. Consequently, a single circulating glucocorticoid signal can simultaneously generate distinct—but physiologically coordinated—responses in immune cells, vascular endothelium, epithelial barriers, metabolically active tissues, and the nervous system, thereby allowing organism-wide adaptation while preserving tissue-specific functional specialization [45,75,82,83]. Recent cross-tissue transcriptomic and systems-level studies further support this concept by demonstrating that GRα orchestrates tissue-selective transcriptional programs across multiple organs rather than eliciting uniform biologic responses [82,84]. Thus, organism-wide coordination by GC–GRα signaling does not require uniform receptor activity or identical transcriptional responses across tissues. Rather, integration may emerge through coordinated heterogeneity, whereby a shared systemic glucocorticoid signal generates distinct yet complementary cell- and tissue-specific adaptive responses in response to local functional requirements and the evolving physiologic context [66,75,76,81].

Another distinguishing biologic property of GC–GRα signaling is its ability to integrate adaptive responses simultaneously across multiple biologic compartments and regulatory timescales. Beyond regulating inflammatory gene expression, GRα coordinates mitochondrial bioenergetics, cellular metabolism, endothelial and epithelial barrier integrity, lymphatic endothelial function and tissue-fluid homeostasis, neuroendocrine function, oxidative stress responses, tissue repair, and inter-organ communication networks. This breadth of regulation enables diverse adaptive processes to function as components of a coordinated organism-wide adaptive system rather than as isolated responses. By linking these biologic systems through a common endocrine signaling pathway, GC–GRα promotes coordinated organism-wide adaptation while allowing individual tissues to execute specialized functions appropriate to their physiologic roles [33,69,75,80,85,86].

Because the adaptive priorities of the organism evolve continuously during severe physiologic stress, the regulatory requirements of homeostatic correction must also change over time. The context-dependent nature of GC–GRα signaling allows adaptive programs to be dynamically recalibrated according to the biologic phase of the response, supporting early host defense during the Priming Phase, controlled immune and metabolic recalibration during the Modulatory Phase, and coordinated tissue repair and physiologic recovery during the Restorative Phase [10,23,87]. Collectively, these biologic properties position GC–GRα as an exceptionally well-suited central integrative regulator capable of coordinating adaptive responses across virtually every organ system while preserving the tissue-specific specialization required for effective homeostatic correction. Together, these biologic properties provide the mechanistic foundation for the phase-specific model of homeostatic correction proposed in this review.

## 5. Coordinated Integration of Organ-System Adaptive Programs

The preceding sections demonstrate that successful homeostatic correction requires coordinated activation, integration, and dynamic recalibration of multiple adaptive biologic programs rather than the independent function of individual organs or isolated signaling pathways. Current evidence increasingly supports this systems-level perspective, demonstrating that severe physiologic stress elicits a dynamic, organism-wide adaptive response in which immune, neuroendocrine, metabolic, vascular, mitochondrial, epithelial, lymphatic, microbiome, and regenerative systems communicate continuously through interconnected signaling networks [33,45,50,88].

Rather than operating as parallel, autonomous systems, these adaptive programs are functionally integrated through bidirectional communication mediated by endocrine hormones, autonomic and sensory neural pathways, cytokines, chemokines, extracellular vesicles, metabolites, endothelial and lymphatic signaling, and microbiome-derived mediators. These distributed communication networks continuously integrate and exchange information regarding pathogen burden, tissue injury, metabolic status, oxygen availability, vascular integrity, microbial composition, and regenerative requirements, thereby allowing adaptive responses to be proportionally adjusted as physiologic conditions evolve [89,90,91,92,93].

This systems-level organization enables the organism to function as a coordinated adaptive network in which multiple biologic systems continuously exchange information and adjust their activity according to evolving physiologic demands rather than operating as independently regulated organs. During the Priming Phase, these communication networks rapidly mobilize host-defense, metabolic, neuroendocrine, and vascular-support programs to maximize survival. During the Modulatory Phase, they facilitate coordinated immune recalibration, disease tolerance, and restoration of physiologic stability. During the Restorative Phase, they support inflammatory resolution, tissue repair, regenerative remodeling, and re-establishment of long-term homeostasis. Because these adaptive priorities evolve continuously throughout severe physiologic stress, effective homeostatic correction requires ongoing communication and synchronization among all participating biologic systems rather than sequential activation of isolated pathways [50,88,94,95].

Although these adaptive programs are presented separately for conceptual clarity, they operate simultaneously, interact continuously through multiple communication networks, and together determine the effectiveness of homeostatic correction. Within this conceptual model, the following sections examine the principal adaptive programs involved in homeostatic correction and illustrate how their dynamic interactions coordinate organism-wide adaptation, preserve physiologic resilience, promote recovery, and ultimately restore systemic homeostasis.

## 6. Functional Roadmap of the Adaptive Programs

The preceding sections establish the biologic rationale for homeostatic correction as a coordinated, phase-specific process requiring continuous integration among multiple adaptive biologic systems. Table 2 summarizes the principal adaptive objectives of the major biologic domains across the Priming, Modulatory, and Restorative phases, providing a functional roadmap of homeostatic correction.

Although each biologic domain is presented individually for conceptual clarity, the adaptive programs operate simultaneously and remain continuously integrated through extensive endocrine, neural, immune, metabolic, vascular, lymphatic, mitochondrial, microbial, and regenerative communication networks. The dominant functional priorities of each domain evolve as physiologic conditions change, enabling coordinated progression from adaptive readiness and survival, through physiologic recalibration and stabilization n, to restoration of systemic homeostasis, physiologic resilience, and long-term recovery. Accordingly, the three phases should not be interpreted as fixed, sequential states; rather, they represent partially overlapping, asynchronous biologic programs whose relative dominance may vary across tissues, cell populations, and organ systems. Experimental and clinical evidence supports the individual molecular and physiologic mechanisms within the framework to varying degrees, whereas their organization into phase-specific, organism-wide integrative relationships is a proposed conceptual model that requires prospective validation.

The following sections summarize the principal adaptive programs that constitute the framework of homeostatic correction. Their underlying mechanisms are examined in greater detail in two complementary companion reviews: Companion Manuscript 1 [24] examines the foundational immune, metabolic, mitochondrial, and vascular adaptive responses, whereas Companion Manuscript 2 [25] examines the complementary neuroendocrine, adaptive immune, lymphatic, microbiome–gut, epithelial barrier, and mechanical–structural regenerative responses. Together, these companion reviews describe the integrated biologic systems that support adaptive capacity, physiologic resilience, and recovery across the Priming, Modulatory, and Restorative Phases.

Operational identification of these phases will likely require longitudinal assessment of multidomain biologic trajectories rather than reliance on a single biomarker or fixed threshold. Candidate features of the Priming Phase include activation of innate immune and acute-phase responses (e.g., leukocyte activation, circulating inflammatory mediators, C-reactive protein), HPA-axis activation, metabolic substrate mobilization, endothelial activation, and early mitochondrial stress. The Modulatory Phase may be characterized by shifting trajectories of inflammatory and acute-phase markers, along with measures of immune competence or dysfunction (e.g., monocyte HLA-DR expression, lymphocyte counts and function), endothelial-barrier function, metabolic and mitochondrial recovery, and neuroendocrine recalibration. The Restorative Phase may be reflected by sustained resolution of inflammatory activity, recovery of innate and adaptive immune competence, restoration of endothelial and metabolic homeostasis, tissue-repair and regenerative activity, and progressive recovery of organ and physiologic function. Importantly, these candidate features are proposed for prospective evaluation rather than as validated diagnostic criteria, and discordant trajectories among biologic domains may themselves identify asynchronous or impaired homeostatic correction.

## 7. Scientific and Clinical Implications

The homeostatic correction model provides a systems-level perspective for understanding how the organism adapts to severe physiologic stress and critical illness [32]. Rather than viewing immune, metabolic, neuroendocrine, vascular, mitochondrial, lymphatic, microbiome, and reparative responses as independent biologic processes, the model recognizes these responses as interdependent, coordinated adaptive programs whose dynamic integration determines the effectiveness of host defense, physiologic resilience, and recovery [96,97,98]. By emphasizing coordinated regulation across multiple organ systems [99], the model offers a conceptual foundation for integrating diverse observations from basic, translational, and clinical research into a unified model of organism-wide adaptation [33].

### 7.1. Scientific Implications

This model has several important implications for future investigation. First, it supports a shift from reductionist models that examine individual organs or isolated signaling pathways toward systems-level analyses that investigate coordinated interactions among adaptive biologic systems [32,100,101]. Increasing evidence indicates that homeostatic correction emerges from dynamic communication among immune, neuroendocrine, metabolic, vascular, mitochondrial, lymphatic, microbiome, and regenerative networks [33,98,99]. Rather than from the independent function of individual organs or isolated signaling pathways [97,102]. Understanding these coordinated interactions may therefore provide a more comprehensive explanation for physiologic adaptation, resilience, and recovery during severe physiologic stress and critical illness [33].

The three-phase organization of homeostatic correction also provides a biologically structured model for understanding how adaptive responses evolve during severe physiologic stress [23,33]. Rather than viewing host responses as static or dichotomous (e.g., pro- versus anti-inflammatory), homeostatic correction proceeds through sequential yet overlapping Priming, Modulatory, and Restorative phases, each with distinct yet highly coordinated biologic priorities [10,23]. This temporal organization provides a biologically grounded model for investigating how immune, neuroendocrine, metabolic, vascular, mitochondrial, lymphatic, microbiome, and reparative programs are activated, coordinated, and ultimately restored during the adaptive response [101,102,103]. By linking biologic function to the timing and phase of illness, this model offers a dynamic perspective on physiologic adaptation, resilience, and recovery [32,33].

Finally, this model provides a foundation for integrating systems biology, multi-omics technologies, computational modeling, and artificial intelligence to investigate adaptive biologic networks and to develop quantitative measures of adaptive capacity [104,105,106]. Because adaptive responses arise from dynamic interactions among genes, proteins, metabolites, cells, tissues, and organ systems, integrated analytical approaches are increasingly recognized as essential for understanding the complexity of homeostatic regulation [107,108,109]. By integrating molecular, physiologic, and clinical data, these technologies have already demonstrated substantial potential to improve mechanistic understanding, identify multidimensional biomarkers, characterize disease subtypes, and predict biologic trajectories [110,111]. Although further standardization, interpretability, and prospective clinical validation are still necessary before widespread implementation [112], these integrative approaches provide a promising foundation for developing dynamic biomarkers and precision strategies to assess adaptive capacity and phase-specific homeostatic correction across acute and chronic disease states [33].

### 7.2. Clinical Implications

The homeostatic correction model also has important implications for clinical investigation and for the future development of precision medicine. Viewing critical illness and many chronic diseases as disorders of disrupted homeostatic correction, rather than as isolated abnormalities in individual organs, provides a biologically organized model for evaluating the integrated adaptive responses that determine physiologic resilience, recovery, and clinical outcome [32,33,101].

This systems-level perspective supports moving beyond single biomarkers toward multidomain assessment of adaptive capacity. Rather than relying on isolated inflammatory, endocrine, or metabolic measurements, future assessment strategies may integrate biomarkers that reflect immune competence, neuroendocrine regulation, mitochondrial function, endothelial integrity, metabolic adaptation, tissue repair, and inter-organ communication to provide a more comprehensive evaluation of the organism’s adaptive state [38,99,106,113].

Such multidomain assessment may also be important for interpreting therapeutic response. Because critical illness encompasses substantial biological heterogeneity, patients with similar clinical presentations may have different underlying biological states and respond differently to the same treatment [114]. Recent ARDS studies illustrate this principle: inflammatory and proteomic phenotypes were associated with different responses to corticosteroid therapy, suggesting that treatment effects may depend on the host’s evolving biological state [57,115]. These findings support investigating treatments tailored to the patient’s evolving biological state, but prospective validation is needed before clinical implementation.

Combining multidomain biologic data with computational modeling and artificial intelligence may help identify the patient’s current phase of homeostatic correction, assess adaptive capacity, and track changes in the biologic response over time. These approaches may ultimately help clinicians monitor treatment response, identify patients at risk of poor recovery, and select therapies appropriate to the patient’s evolving biological state. However, prospective studies are needed before clinicians can incorporate these strategies into practice [33,112,116,117].

More broadly, because disruption of coordinated adaptive responses is a shared feature of many acute and chronic disorders, the homeostatic correction model may provide a common biologic foundation for developing phase-specific diagnostic, prognostic, and therapeutic approaches across diseases traditionally considered unrelated. By focusing on restoring adaptive capacity rather than treating isolated abnormalities, this systems-level perspective supports the development of precision medicine strategies to restore coordinated adaptive function and improve long-term patient outcomes [33,102].

## 8. Conclusions

Severe physiologic stress and critical illness are increasingly recognized as states of disrupted systemic homeostasis rather than simply consequences of maladaptive systemic inflammation or isolated organ failure. Survival and recovery depend on the coordinated activation, recalibration, and resolution of adaptive biologic programs spanning immune, neuroendocrine, metabolic, mitochondrial, vascular, epithelial, lymphatic, microbiome, and regenerative systems.

The phase-specific homeostatic correction model offers a biologically organized model for understanding how these interconnected adaptive responses evolve during severe physiologic stress. The Priming Phase establishes adaptive readiness and immediate survival responses; the Modulatory Phase limits excessive activation while preserving essential host-defense and physiologic functions; and the Restorative Phase promotes resolution, tissue repair, regenerative recovery, and restoration of integrated organ-system function. Although presented sequentially for conceptual clarity, these phases are dynamic, overlapping, and continuously coordinated across temporal, spatial, and tissue-specific contexts.

In this model, GC–GRα signaling serves as a central integrator of organism-wide adaptation. Through context-dependent interactions with immune, metabolic, mitochondrial, vascular, neuroendocrine, epithelial, lymphatic, microbiome, and regenerative pathways, GC–GRα signaling helps coordinate inter-organ communication, disease tolerance, tissue protection, and recovery while preserving essential host-defense functions. Failure to appropriately activate, coordinate, transition between, or terminate these adaptive programs may contribute to persistent inflammation, immune dysfunction, metabolic and mitochondrial abnormalities, endothelial injury, impaired repair, chronic critical illness, and progressive loss of adaptive resilience. Accordingly, improved understanding of phase-specific homeostatic correction and the mechanisms regulating GC–GRα signaling may provide the biologic foundation for developing phase-specific biomarkers, identifying novel therapeutic targets, and advancing precision strategies to restore long-term physiologic resilience.

Extensive experimental and clinical evidence supports the individual biologic processes comprising the Priming, Modulatory, and Restorative phases, along with the regulatory actions of GC–GRα signaling within them. The novelty of the present model lies not in proposing previously unrecognized biologic mechanisms, but in integrating established, historically compartmentalized observations into a unified, phase-specific model in which context-dependent GC–GRα signaling helps coordinate organism-wide adaptation from initial host defense through physiologic recalibration to the restoration of homeostasis. Accordingly, the integrated model is a testable conceptual construct whose validation will require prospective longitudinal evaluation of GC–GRα function alongside multidomain biologic trajectories and phase transitions.

The homeostatic correction model provides a biologically grounded foundation for investigating adaptive capacity and for developing precision approaches to evaluate, monitor, and restore coordinated adaptive function across acute and chronic disease states. This framework may also guide the development of phase-specific biomarkers and quantitative measures of adaptive capacity for future use in precision medicine. The two companion manuscripts [24,25] extend this conceptual model by examining the underlying phase-specific adaptive programs and their coordinated roles in organism-wide homeostatic regulation. Collectively, this body of work marks a shift from viewing critical illness primarily as a disorder of maladaptive systemic inflammation or isolated organ dysfunction to understanding it as a disorder of disrupted organism-wide adaptive coordination, thereby providing a conceptual foundation for phase-specific homeostatic correction.

## Figures and Tables

**Table 1:** Biological and Mechanistic Properties Supporting GC–GRα as a Central Integrative Regulator of Homeostatic Correction.

Distinguishing Property	Mechanistic Basis	Why It Supports a Central Integrative Role	Key Supporting Evidence
**Evolutionary/developmental integration**	Conserved vertebrate corticosteroid signaling; roles in development, maturation, reproduction and survival	Establishes GC–GRα as a fundamental adaptive system across the life cycle	Primary evolutionary/developmental studies [3]
**Organism-wide cellular accessibility**	Systemic GC delivery and GRα expression across virtually all nucleated cells	Allows a common neuroendocrine stress signal to reach essentially the entire organism	Distribution and expression studies [74]
**Coordinated heterogeneity**	Chromatin accessibility, cofactors, receptor state and local signaling generate tissue-specific responses	Explains how one systemic signal produces distinct but complementary responses across tissues	Cross-tissue and chromatin studies; [75,76]
**Nuclear–mitochondrial integration**	GR regulates nuclear transcription and mitochondrial function	Couples adaptive transcription responses to cellular bioenergetic requirements	Nuclear- mitochondrial-GR studies [69,80]
**Network crosstalk**	Interaction with NF-κB, AP-1, MAPK, STAT, HIF, and other signaling networks	Allows GR to integrate with—rather than replace—other major adaptive regulators	Mechanistic signaling studies [76,77]
**Multisystem/inter-organ integration**	Regulation of immune, metabolic, vascular, neuroendocrine, epithelial, lymphatic, and other adaptive systems and their inter-organ communication	Provides an architecture for organism-wide coordination across specialized tissues and organ systems	Tissue-specific GR and inter-organ studies [33]
**Dynamic/context-dependent regulation**	GR abundance, isoforms, phosphorylation, translocation, cofactors and ligand availability change with context	Allows biological output to change as adaptive requirements evolve	Receptor regulation and human critical-illness studies [66,74,78]
**Feedback and continuous recalibration**	HPA–GC–GR feedback and peripheral regulation	Provides a mechanism for adjusting magnitude and duration of the stress response	HPA/GR feedback studies [79]

Abbreviations: AP-1, activator protein 1; GC, glucocorticoid; GR, glucocorticoid receptor; GRα, glucocorticoid receptor alpha; HIF, hypoxia-inducible factor; HPA, hypothalamic–pituitary–adrenal; MAPK, mitogen-activated protein kinase; NF-κB, nuclear factor kappa B; STAT, signal transducer and activator of transcription.

**Table 2:** Functional Roadmap of Homeostatic Correction Across the Priming, Modulatory, and Restorative Phases.

Biologic Domain	Priming Phase (Ready–Reinforce)	Modulatory Phase (Recalibrate–Stabilize)	Restorative Phase (Resolve–Restore)
Immune–Inflammatory	Detect danger signals; activate innate host defense; recruit immune cells	Recalibrate concurrent inflammatory and immunosuppressive responses; preserve antigen presentation and lymphocyte function; limit maladaptive regulatory expansion and immune exhaustion while maintaining host defense	Resolve inflammation; restore immune competence and homeostasis; recover effective adaptive responses; establish immunologic memory and durable host protection
Neuroendocrine	Activate HPA axis and stress responses; support survival adaptation	Restore feedback regulation; rebalance autonomic function; limit persistent stress activation	Re-establish neuroendocrine homeostasis; support cognition, recovery, and physiologic resilience
Metabolic–Bioenergetic	Mobilize glucose, lipids, and amino acids; generate ATP; support acute energy demands	Improve mitochondrial efficiency; restore metabolic balance; reduce oxidative stress	Restore metabolic flexibility; normalize substrate utilization; support long-term bioenergetic recovery
Mitochondrial	Increase bioenergetic output; support stress adaptation and host defense	Recover mitochondrial integrity; enhance quality control; restore redox balance	Re-establish mitochondrial homeostasis; support tissue regeneration and organ recovery
Vascular–Endothelial	Promote leukocyte recruitment; maintain perfusion and vascular responsiveness	Stabilize endothelial barrier function; reduce permeability; rebalance coagulation	Repair endothelial structure; restore glycocalyx integrity; normalize microcirculatory function
Adaptive Immunity	Initiate antigen presentation and adaptive immune activation	Limit excessive T-cell activation; promote immune regulation	Restore adaptive immune homeostasis; maintain immune competence and memory
Lymphatic	Enhance fluid drainage and immune-cell trafficking	Improve inflammatory mediator clearance; support lymphatic remodeling	Restore lymphatic architecture, flow, and coordinated immune-cell trafficking
Microbiome–Gut	Preserve barrier integrity; limit microbial translocation	Restore epithelial function; support beneficial microbial communities	Re-establish microbiome diversity, microbial metabolite production, and gut–immune–brain communication
Mechanical–Structural & Regenerative	Limit tissue injury; preserve structural integrity	Promote controlled tissue repair; regulate ECM remodeling; limit fibrosis	Restore organ architecture, muscle function, tissue regeneration, and functional recovery
Integrated Homeostatic Goal	Immediate survival and adaptive readiness	Physiologic stabilization and controlled recovery	Restoration of systemic homeostasis, resilience, and long-term recovery

Abbreviations: ATP = adenosine triphosphate; ECM = extracellular matrix; HPA = hypothalamic–pituitary–adrenal.

## Data Availability

No new data were created or analyzed in this study. Data sharing is not applicable to this article.
